# Improving the culture surrounding tracheal aspirates in mechanically ventilated children

**DOI:** 10.1128/jcm.00109-26

**Published:** 2026-06-12

**Authors:** Monika Jelic, Matthew Weber, Meghan Birkholz, Sarah Parker, Stacey Hamilton, Andrea Prinzi, Kellie Rusin, Sarah Jung, Christopher D. Baker, Samuel R. Dominguez

**Affiliations:** 1 Department of Pediatrics, University of Colorado School of Medicine 12225, Aurora, Colorado, USA; 2 Children’s Hospital Colorado, Aurora, Colorado, USA; 3 University of Colorado Clinical and Translational Sciences Institute 513712, Aurora, Colorado, USA; Children's Hospital Los Angeles, Los Angeles, California, USA

**Keywords:** tracheal aspirates, quality improvement, diagnostic stewardship

## Abstract

Interpretation of tracheal aspirate cultures is limited by contamination with normal respiratory flora and inconsistency in collection, processing, and reporting. Our aim was to both decrease waste and improve the quality and interpretability of tracheal aspirate cultures by implementing laboratory-driven Gram stain rejection criteria. We reviewed prior tracheal aspirate specimens and cultures, examined the current literature, and gathered input from stakeholders to inform potential Gram stain rejection criteria. Rates of tracheal aspirates collected, cultured, and rejected pre-intervention (9 November 2019–2 April 2023) and post-intervention (3 April 2023–28 February 2024) were compared. We implemented culture rejection criteria and rejected specimens with Gram stains demonstrating moderate to heavy epithelial cells and/or no organisms/three or more organism morphologies. The median rate of tracheal aspirates cultured decreased from 7.7 to 2.4 per 100 ventilator days (*P* < 0.001) in the pre- and post-intervention periods, respectively. There was a corresponding decrease in the median proportion of cultures with no growth (15% vs 2.8%, *P* < 0.001) and those growing normal respiratory flora (45% vs 33% per 100 ventilator days, *P* < 0.001) and an increase in actionable cultures with growth of predominant organisms (39%–61%, *P* < 0.001). Implementation of Gram stain rejection criteria led to a 69% decrease in the rate of tracheal aspirate samples cultured and a subsequent 22% increase in cultures with actionable results. This intervention saved laboratory resources and, anecdotally, increased laboratorian satisfaction. Taken together, this initiative increased the value of care.

**IMPORTANCE:**

Tracheal aspirate cultures are often limited by contamination with upper respiratory flora, resulting in low-yield information, unnecessary laboratory utilization, and potential misinterpretation that may influence antimicrobial use. We demonstrate that implementation of standardized, laboratory-driven Gram stain rejection criteria significantly reduces the number of cultures performed while increasing the proportion of clinically actionable results. This diagnostic stewardship intervention improved the interpretability of tracheal aspirate cultures, decreased waste, and enhanced the overall value of care. Our findings provide a practical, scalable framework for clinical microbiology laboratories to optimize tracheal aspirate culture workflows and better align testing with meaningful clinical outcomes.

## INTRODUCTION

Tracheitis and ventilator-associated pneumonia are difficult to diagnose in children since the Centers for Disease Control and Prevention criteria are not validated in children, and cultures are neither specific nor sensitive (1). For ventilated patients, tracheal aspirate (TA) cultures are routinely used in conjunction with clinical criteria to diagnose lower respiratory tract infections. However, the accuracy of TA cultures for this diagnostic purpose is low and limited since the airway is not sterile and cultures may be impacted by significant contamination with normal respiratory flora and colonizing organisms, making interpretation difficult (2). Additionally, due to inconsistency in collection, processing, and reporting, many of these cultures are not clinically interpretable and contribute to practice variation. Despite published culture processing and interpretation recommendations from the American Society of Microbiology (3), a nationwide study demonstrated that reporting of TA Gram stains and culture results is highly variable (4). Additionally, 23% (14/61) of clinical microbiology laboratories surveyed used rejection criteria based on Gram stain (4), thus implementation is within the scope of standard practice.

Importantly, reported “positive” cultures often directly impact clinical decision-making to start and/or continue antibiotics, likely resulting in excess antibiotic use (5, 6). Unfortunately, excess antibiotic use in this critically ill population can lead to unintended downstream complications, including increased risk of *Clostridiodies difficile* infections, development of colonization and infection with drug-resistant organisms, higher incidence of adverse drug reactions, microbiome changes, and increased hospital costs (7). Culturing poor-quality TA specimens leads to suboptimal clinical care at more cost and thus lower value. To address these issues, diagnostic stewardship attempts have primarily targeted interventions at the provider level with clinical decision support tools. These interventions have had limited success in some settings, with variability in sustainability (7, 8).

Our aim was to improve the quality and interpretability of TA cultures by implementing laboratory-driven Gram stain rejection criteria without adversely impacting clinical care. By only culturing high quality specimens and those with significant bacterial burden, our goal was to decrease culturing of low-value samples and increase the percentage of cultures with actionable results, thus improving care value.

## MATERIALS AND METHODS

### Development of rejection criteria and setting

Children’s Hospital Colorado (CHCO) is a ~500-bed quaternary care, free-standing children’s hospital, serving a seven-state region. There are 82 neonatal intensive care unit (NICU) beds, 22 cardiac intensive care unit (CICU) beds, and 48 pediatric intensive care unit (PICU) beds. We have an active antimicrobial stewardship program that has practiced handshake stewardship since 2013 (9, 10) and an on-site microbiology laboratory. An initial review of TA specimens was completed at CHCO in 2018 summarizing Gram stain results and culture growth. A multidisciplinary team consisting of clinical microbiologists, infectious disease physicians, a pulmonologist, and antimicrobial stewards reviewed these data and gathered input from stakeholders. To determine culture rejection criteria, varying combinations of Gram stain results (polymorphonuclear cells [PMNs], epithelial cells, and organisms) were evaluated to determine which yielded clinically actionable culture data (predominant growth of one or two organisms). We elected not to include PMNs in our rejection criteria, as there was a lack of correlation between the quantity of PMNs on Gram stain and culture growth. Additionally, as we care for a large immunocompromised patient population, stakeholders did not want cultures to be rejected by patients with underlying neutropenia. For epithelial cells, which are more indicative of oral secretions, we elected to use the already established rejection criteria for sputum (11). Finally, when reviewing our data for the correlation between organisms seen on Gram stain and subsequent culture results, those with no organism only had a pure culture with a respiratory pathogen in 5% of specimens, and specimens with three or more organisms on Gram stain grew normal upper respiratory flora (three or more organisms on culture) in 98% of specimens. Thus, we elected to reject those low-value specimens. In summary, TA specimens would not be cultured if the Gram stain demonstrated

1. Moderate (10–25 cells per single or high-power field at 40×) or heavy (>25 cells) epithelial cells.
2. No organisms on Gram stain.
3. Three or more different Gram stain morphologies.

Proposed rejection criteria were presented to high-use stakeholders, including providers in the PICU, CICU, NICU, the inpatient pulmonary service, and the emergency department for critical review and input starting January 2023. Our target goal was to decrease overall culturing of tracheal aspirates by 20%.

### Intervention implementation

Prior to implementation, microbiology leadership instituted standardized training for all staff that included reviewing multiple training slides for each of the potential Gram stain possibilities. Once implemented, there was ongoing quality assessment to compare culture results with Gram stain findings to evaluate ongoing concordance. Rejection criteria were implemented on 3 April 2023 and were applied to all TA specimens collected and processed in the CHCO Microbiology Laboratory. When a specimen did not meet culture criteria, a comment was included with the Gram stain results stating the specimen would not be cultured and the reason why it was rejected. The results also informed the ordering provider that they could call the microbiology laboratory within 48 hours to request a culture. As a laboratory balancing measure, we monitored the number of requests for culture on rejected specimens and the subsequent growth of these rejected, then cultured samples. As a clinical balancing measure, we monitored the median ventilatory days per patient pre- and post-intervention in the PICU.

### Data collection

Data on TA specimens were compared between two time periods. Our pre-intervention time frame was 9 November 2019–1 April 2023, and our post-intervention time frame was 3 April 2023–28 February 2024. TA specimen data and patient demographics were obtained from our internal electronic medical records (EMRs) and included specimens collected from endotracheal tubes as well as tracheostomies, since these could not be reliably differentiated within the EMR data. Specimen-level data included Gram stain information, culture growth, collection date, and department. Patient-level data included demographics, admission age, insurance type, race, and ethnicity. Ventilator days and patient unit locations were obtained from daily charge data pulled from the Pediatric Hospital Information Systems database and linked to our internal EMR data. To estimate the cost associated with the processing of tracheal aspirate cultures, we used $180 per sample, which included supplies, labor, organism identification, and susceptibility testing. To evaluate the change in tracheitis incidence pre- and post-intervention, we used the National Healthcare Safety Network (NHSN) 2014 definition for tracheitis and standardized the rate per 100 ventilated days (12). Due to discontinuation of routine surveillance at our institution for tracheitis before the intervention and to account for potential seasonal variation, we chose a similar 6-month monitoring period (June–November) pre- and post-intervention for comparison of the tracheitis outcome.

### Analysis

Comparisons were made between the pre-intervention group, in which all TA specimens were cultured, and the post-intervention group; the post group contained specimens that were not cultured due to meeting rejection criteria, specimens that were cultured due to meeting criteria, and specimens that were initially rejected but were cultured due to clinician request. Specimens that were cultured at clinician request, despite being initially rejected, were excluded from analysis of culture rate and proportion of cultures growing normal respiratory flora or having no growth. Ten percent of the TA specimens in the post-implementation group were reviewed for confirmation of Gram stain results and correct application of the rejection criteria. Statistical process control U charts were used to identify changes in the rate of specimen collection and culture per 100 ventilator days measured over the study period. P charts were created to present the proportion of cultures growing either nothing or normal respiratory flora. Standard rules were used to identify special cause variation and calculate upper and lower control limits (13). As an additional measure of the intervention, a Mann-Whitney test was performed to confirm the results for all outcomes before and after the intervention. Both parametric and non-parametric tests were completed and had concordant results, though only non-parametric results are presented after verifying that the outcomes likely did not meet the assumptions for a *t*-test. All data presented in the tables were tested using the two-tailed chi-square method. *P* values of less than 0.05 were considered statistically significant.

## RESULTS

### Tracheal aspirates collected and cultured

In the 41-month pre-intervention period, 4,206 TA specimens were collected, while in the 11-month post-period, 937 were collected. Most specimens were sent from the PICU, pre- and post-intervention. Table 1 shows the demographics of the unique patients who had TA specimens collected, and Table 2 includes the specimen-specific results pre- and post-intervention.

**TABLE 1:** Tracheal aspirate specimen demographics for unique patients

	Pre-intervention 11/9/2019–4/2/2023^*c*^ *N* = 1,515	Post-Intervention 4/3/2023–2/28/2024^*c*^ *N* = 429	*P* value^*a*^
Median age at admission (years)	1.76	1.82	0.6758
Female, *n* (%)	666 (44)	197 (45.9)	0.4706
Race (first listed)			
White/Caucasian	1,005 (66.3)	295 (68.8)	0.3456
Other	161 (10.6)	51 (11.9)	0.4595
Black/African American	110 (7.3)	52 (12.1)	0.0013
Unknown^*b*^	115 (7.6)	8 (1.9)	0.0001
Asian	55 (3.6)	9 (2.1)	0.1163
American Indian/Alaska Native	43 (2.8)	9 (2.1)	0.4015
Native Hawaiian/other Pacific Islander	8 (0.5)	5 (1.2)	0.1527
More than one race	18 (1.2)	0 (0)	0.0191
Ethnicity			
Not Hispanic or Latino	935 (61.7)	265 (61.8)	0.9834
Hispanic or Latino	467 (30.8)	153 (35.7)	0.0576
Unknown^*b*^	113 (7.5)	11 (2.6)	0.0003
Insurance			
Medicaid	856 (56.5)	258 (60.1)	0.1787
Contract/private	572 (37.8)	150 (35)	0.2909
Tricare	52 (3.4)	12 (2.8)	0.5151
Other	35 (2.3)	9 (2.1)	0.7941

^*a*^ *P* values were obtained using chi-squared/Fisher's exact test and were derived by comparing observed and expected counts within each demographic variable, assuming no difference in their distribution between the two intervention groups.

^*b*^ Category reported as unknown, not reported, or declined to answer.

^*c*^ Denotes mo/day/year.

**TABLE 2:** Tracheal aspirate specimen data^*a*^

	Pre-Intervention 11/9/2019–4/2/2023^*g*^ *N* = 4,206^*b*^	Post-Intervention 4/3/2023–2/28/2024^*g*^ *N* = 937^*b*^	*P* value^*c*^
Collection department, *n* (%)			
PICU	2,068 (49.2)	378 (40.3)	0.0001
NICU	908 (21.6)	234 (25.0)	0.0242
CICU	545 (13.0)	128 (13.7)	0.5639
Other, non-ICU	685 (16.3)	197 (21.0)	0.0005
TA, *n* (%)			
Rejected, not cultured	NA	507 (54.1)	NA
Total cultured	4,206 (100)	430 (45.9)	0.0001
Met culture criteria	NA	398 (92.6)	NA
Rejected, culture requested	NA	32 (7.4)	NA
TA rejected, cultured requested			
Moderate/heavy epithelial cells	NA	0^*d*^	NA
No organisms	NA	2	NA
3+ organisms	NA	30	NA
Organism morphologies on Gram stain, *n* (%)			
None^*f*^	1,663 (40.0)	293 (31.3)	0.0001
1	1,136 (27.0)	240 (25.6)	0.3829
2	755 (18.0)	158 (16.9)	0.4305
3^*f*^	460 (10.9)	157 (16.8)	0.0001
4^*f*^	163 (3.9)	71 (7.6)	0.0001
5^*f*^	26 (0.6)	18 (1.9)	0.0001
6^*f*^	3 (0.1)	0 (0)	~ 1
PMNs on Gram stain, *n* (%)			
None	385 (9.2)	39 (4.2)	0.0001
Rare	1,220 (29.0)	261 (27.9)	0.4815
Few	1,051 (25.0)	285 (30.4)	0.0006
Moderate	930 (22.1)	204 (21.8)	0.8206
Heavy	618 (14.7)	145 (15.5)	0.5427
NA	2 (0.1)	3 (0.3)	0.0451
Epithelial cells on Gram stain, *n* (%)			
None	2,859 (68.0)	572 (61.1)	0.0001
Rare	1,168 (27.8)	316 (33.7)	0.0003
Few	140 (3.3)	37 (4.0)	0.3463
Moderate^*f*^	13 (0.3)	2 (0.2)	~1
Heavy^*f*^	1 (0.1)	1 (0.1)	0.3312
NA	25 (0.6)	9 (1.0)	0.2111
Culture result, *n* (%)^*e*^			
MURF (3+ org)	1,924 (45.7)	144 (36.2)	0.0001
No growth	648 (15.4)	10 (2.5)	0.0001
One or two predominant organisms	1,634 (38.9)	244 (61.3)	0.0001
One predominant organism	1,223 (29.1)	153 (38.4)	0.0001
Two predominant organisms	411 (9.8)	91 (22.9)	0.9554

^*a*^ ICU, intensive care unit; MURF, mixed upper respiratory flora; NICU, neonatal intensive care unit; PICU, pediatric intensive care unit; PMN, polymorphonuclear cell; TA, tracheal aspirate; NA, not applicable.

^*b*^ Total specimens collected.

^*c*^ *P* values were obtained using the chi-squared/Fisher's exact test and were derived by comparing observed and expected counts within each demographic variable, assuming no difference in their distribution between the two intervention groups.

^*d*^ Also rejected based on organism Gram stain results.

^*e*^ Out of only specimens meeting culture criteria, *N* = 398 post-intervention.

^*f*^ Meets rejection criteria, not cultured.

^*g*^ Denotes mo/day/year.

**Fig 1:**
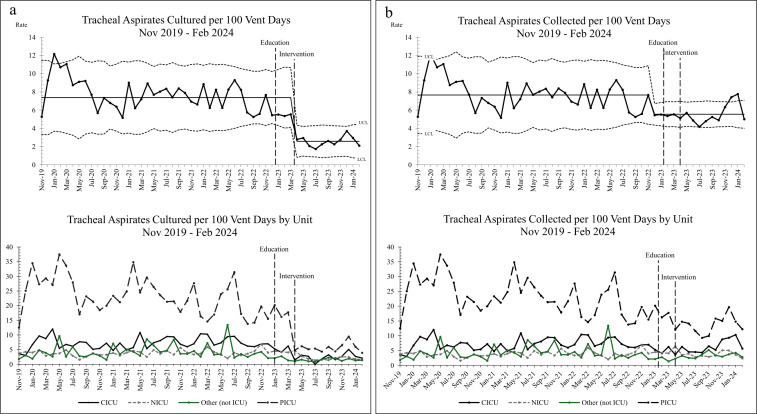
Rate of tracheal aspirates cultured (**a**) and collected (**b**) per 100 vent days per unit by month.

**Fig 2:**
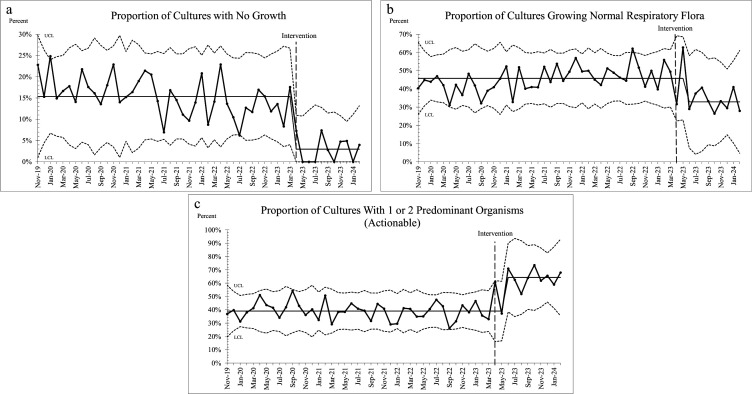
Proportion of tracheal aspirate cultures with no growth (**a**) or those with three or more organisms (**b**) or actionable cultures (one or two organisms on culture) (**c**) per month pre- and post-intervention of all cultured specimens.

Prior to the intervention, all collected TA specimens were cultured; post-intervention, 45.9% were cultured (42.5% met criteria for culture, 3.4% were initially rejected then cultured at the ordering provider’s request). There was special cause variation with a decrease in the median rate of cultured TA from 7.7 to 2.4 per 100 vent days (*P* < 0.001) in the pre- and post-intervention period, respectively. For TA collected, there was a decrease from a median rate of 7.7 to 5.1 per 100 vent days (*P* < 0.001) (Fig. 1). We also found special cause variation in the percentage of cultures with no growth, with growing normal respiratory flora, and with clinically actionable results (Fig. 2). Pre-intervention, a median of 15% of cultures had no growth, and post-intervention, this decreased to 2.8% (*P* < 0.001). For cultures growing normal respiratory flora, there was a median proportion of 45% pre-intervention cultures and 33% cultures post-intervention (*P* < 0.001). The median proportion of cultures with clinically actionable results (growth of one or two predominant organisms) increased from 39.8% to 62.5% after rejection criteria were implemented (*P* < 0.001). Our results were the same when we compared the post-intervention period to a shorter pre-intervention interval during April 2022–February 2023.

### Rejected tracheal aspirate specimens

In the 11-month post-intervention period, 58% of specimens were rejected based on the established Gram stain criteria. Of these rejected specimens, 32/539 (6%) were subsequently cultured by provider request, including 2 with no organisms on Gram stain and 30 with three or more Gram stain morphologies. Of the specimens initially rejected and subsequently cultured, 6/32 (18%) subsequently grew only one or two predominant organisms.

### Balancing and value measures

The median pre-intervention rate of NHSN-defined tracheitis was 6.5 events per 100 ventilator days compared to a post-intervention rate of 2.0 events per 100 ventilator days (*P* = 0.001). There was no difference in the median per patient ventilatory days pre- and post-intervention for patients in the PICU (3 vs 2 days, *P* = 0.672). The number of TA specimens collected per 100 ventilated days also decreased over time (Table 2).

The median number of ventilated days per month in the post-intervention period was 1,558. Assuming a total cost of $180 per culture, this saves an estimated $14,863.33 per month for an annual cost savings of $178,359.96. Biohazardous waste disposal costs were not included in the cost-saving calculations.

## DISCUSSION

Implementation of laboratory-based TA specimen rejection criteria resulted in a 69% decrease in specimens being cultured, with a greater than threefold reduction in the rate of cultures per 100 ventilator days. Specimens that were cultured were 22.7% more likely to result in actionable data (one or two predominant organisms) and less likely to be negative or grow normal flora. This was one of our primary objectives. On balance, this resulted in decreased ventilator-associated tracheitis rates and did not prolong median ventilatory days for patients in the PICU. Furthermore, the reduction in cultures resulted in significant cost savings and thus increased value for our patients.

A reduction in challenging-to-interpret results from a culture from a non-sterile site is critical to high-value care. Clinicians are more likely to treat patients with positive cultures, and overreporting of positive cultures, including those with predominately normal respiratory flora, can lead to unnecessary antibiotic use and is the leading cause of inappropriate and avoidable antibiotic use in the ICU setting (1, 14, 15). Although beyond the scope of this initial work, we are currently evaluating the impact of our intervention on overall antibiotic use within our ICUs.

Interestingly, our intervention also resulted in a decrease in samples being collected, suggesting that our intervention may have impacted provider decision-making on when to order TA, limiting them in lower-yield clinical situations. The decrease in collection began prior to our laboratory’s rejection criteria, suggesting that pre-intervention education of providers likely also had an impact.

As a balancing measure, providers were able to call the microbiology laboratory to have a rejected specimen cultured, which occurred only in 6% of rejected specimens, a lower percentage than expected. The majority (80%) of rejected specimens that were subsequently cultured occurred during the first 6 months after implementation, suggesting that as time went on providers became more comfortable with the changes and requested fewer over-riding cultures. Additionally, most of the specimens that were cultured after meeting rejection criteria subsequently grew normal respiratory flora, thereby providing increased confidence in the appropriateness and safety of our intervention.

In contrast to our intervention, previous diagnostic stewardship efforts to reduce the overuse of TA cultures have primarily targeted provider-focused and ordering-focused interventions. Sick-Samuels et al, implemented a TA culture clinical decision support algorithm (not embedded within the electronic medical record) which resulted in a 41% reduction in TA cultures from a baseline of 10.9 to 6.5 cultures per 100 ventilator days (7). Ormbsy et al, similarly demonstrated a decrease in TA cultures from 10.7 to 7.1 cultures per 100 ventilator days by implementing a series of quality improvement initiatives including development of appropriate criteria for ordering a culture, standardization of sample collection, limiting repeat cultures, and creating consensus guidelines for initiation of antibiotics (8). More recently, Chiotos et al, implemented a consensus guideline for obtaining TA cultures that was disseminated via education to providers and linked to the order within the electronic medical record that resulted in a reduction in cultures from 4.6 to 2.0 cultures per 100 ventilator days (16). All of these studies demonstrated safety to their intervention with no increases in mortality, readmission rates, or length of stay. Our study adds to this literature by providing an impactful alternative and potentially complimentary approach to these provider- and order-focused interventions.

In support of our approach, the Bright Testing Stewardship for Antibiotic Reduction collaborative recently released clinical practice consensus statements regarding endotracheal aspirate cultures (EACs) in hospitalized children (17). In particular, there were specific recommendations for specimen collection and result interpretation, noting the airway is not sterile and EAC Gram stain results cannot be used in isolation to diagnose a bacterial lower respiratory tract infection because of poor specificity. Additionally, multiple studies have highlighted the high negative predictive value of Gram stains (18). Furthermore, a recent study demonstrated a 20% rate reduction in positive respiratory cultures through a multimodal stewardship intervention that included gatekeeping access to ordering, preferential use of bronchoalveolar lavage for cultures, and suppression of culture results with minimal alveolar neutrophilia (19). No adverse outcomes were noted, suggesting that, similar to our study, restriction of cultures can be part of a safe and impactful diagnostic stewardship intervention. Finally, our results support the new American Thoracic Society guidelines for the judicious use of TA cultures in children with tracheostomies (20). These studies and consensus guidelines support and are in line with our goal to provide higher-quality specimens, which can make an impact on clinical decision-making.

The success of our intervention relied heavily on close partnership with our antimicrobial stewardship team and regular engagement with key stakeholders during the implementation process. Due to the use of handshake stewardship, our team was able to provide in-person feedback and explanation to ICU providers regarding Gram stain results and rejection criteria and provide education when needed (9, 10). This is a good example of the partnership of antimicrobial and diagnostic stewardship to impact clinical care (21). Furthermore, our intervention was relatively easy to implement and was met with little provider resistance since it did not restrict provider ordering or autonomy and did not require significant changes to the EMR.

As an additional benefit, our intervention anecdotally led to an increase in satisfaction among our microbiology laboratory staff. The specific Gram stain criteria for rejection led to less ambiguity and more consistency in processing specimens, leading to higher satisfaction and decreased frustration for the technologists processing and reporting the samples within the laboratory. These changes also had an impact on cost, with an estimated yearly savings of close to $180,000. Furthermore, this resulted in decreased generation of biohazardous waste and therefore a lower carbon footprint.

There are several limitations to our quality initiation. This was implemented at a single center and therefore might not be generalizable to other settings. We had a shorter post-intervention period compared to our pre-intervention time period, which might not accurately predict future impact. We did not complete a full chart review for patients who had rejected specimens that were later cultured and therefore could not quantitate the impact this had on other aspects of their care or outcomes. Prior QI work, including implementation of an order indication, discussions with key stakeholders, and rounding by our antimicrobial stewardship team, may also have impacted the changes.

### Conclusion

This laboratory-based diagnostic stewardship initiative resulted in a 69% rate reduction in the culture of collected TA specimens, and increased the percentage of cultures with actionable results, without increasing ventilator-associated tracheitis or per-patient ventilatory days. As this also resulted in cost and time savings for the laboratory, this initiative increased the value of care.
